# Antigenic repertoire of *Plasmodium vivax *transmission-blocking vaccine candidates from the Indian subcontinent

**DOI:** 10.1186/1475-2875-10-111

**Published:** 2011-05-02

**Authors:** Surendra K Prajapati, Hema Joshi, Virendra K Dua

**Affiliations:** 1 Genetics and Molecular Biology Laboratory, National Institute of Malaria Research (NIMR), Sector-8, Dwarka, New Delhi 110077, India

## Abstract

**Background:**

Genetic polymorphism is an inevitable component of a multistage infectious organism, such as the malaria parasite. By means of genetic polymorphism, parasite opts particular polymorph and reveals survival advantage. *Pvs25 *and *pvs28 *are sexual stage antigen genes, expressed at the ookinete stage inside the mosquito gut, and considered as potential transmission-blocking vaccine candidates. This study presents sequence variations in two important transmission blocking antigen genes *pvs25 and pvs28 *in the field isolates of *P. vivax *from the Indian subcontinent.

**Methods:**

One hundred microscopically diagnosed *P. vivax *isolates were collected from five geographical regions of India. *Pvs25 and pvs28 *genes were PCR amplified and sequenced to assess sequence variation among field isolates.

**Results:**

A total of 26 amino acid substitutions were observed in Pvs25 (10) and Pvs28 (16) among field isolates of *P. vivax*. Tandem repeat polymorphism observed in *pvs28 *shows 3-6 tandem repeats in the field isolates. Seven and eight novel amino acid substitutions were observed in Pvs25 and Pvs28, respectively in Indian isolates. Comparison of amino acid substitutions suggests that majority of substitutions observed in global isolates were also present in Indian subcontinent. A single haplotype was observed to be major haplotype among isolates of Delhi, Nadiad, Chennai and Panna except in isolates of Kamrup. Further, population comparison analyses suggest that *P. vivax *isolates inhabiting in north-eastern region (Kamrup) were distantly related with the isolates from remaining parts of the country. Majority of the amino acid substitutions observed in Indian isolates were more identical to the substitutions reported from isolates of Thailand and Bangladesh.

**Conclusion:**

Study uncovered many new amino acid substitutions as well as a predominance of single haplotype in Indian subcontinent except in north-eastern region of the country. The amino acid substitutions data generated in this study from different geographical regions of the Indian subcontinent could be helpful in designing a more effective anti-malarial transmission-blocking vaccine.

## Background

Malaria is a life-threatening ancient parasitic disease and causes 250-500 million clinical episodes and nearly one million deaths annually [1]. Among the five human malaria species, *Plasmodium falciparum *is the most severe form, causing malignant malaria globally, while *Plasmodium vivax *is the most widespread species outside Africa, causing huge morbidity, and rarely severe and fatal [2-7]. In general, the biology of *P. vivax *differ with *P. falciparum *in three major way 1) relapse, 2) mild disease, and 3) preference of reticulocytes for invasion.

The ookinete surface proteins of *Plasmodium *are the targets for a transmission-blocking vaccine [8-11]. *Pfs25*, *pfs28 *and their orthologs in *Plasmodium *species infecting to human, primate and rodent share a common conserved structure consisting of four tandem epidermal growth factor like domains (EGF) anchored with parasite surface by a glycosylphosphatidylinositol moiety. The purified proteins Pvs25 and Pvs28 along with adjuvant are capable for generating strong immune response in mice. The immune response against sexual stage antigens impaired development of sexual stage of malaria parasite inside mosquito that supports sexual stage antigens can be a potential transmission-blocking vaccine [12]. However, genetic polymorphism in these vaccine candidates can hamper the efficacy of vaccine. Therefore, identifying genetic variations in sexual stage antigen genes is an important task before designing effective anti-malarial control measures.

India is a vast country contributing up to 77.0% of total malaria cases reported in Southeast Asia [13]. *Plasmodium vivax *infections account for more than 50% of the total malaria cases in India and it is one of the two most prevalent malaria parasite species in India. Genetic polymorphism in transmission-blocking vaccine candidates has been reported from various malaria endemic countries such as South and Central America [14-17], Iran [18], Korea [19], Bangladesh [15] and Southeast Asia [15,20] except from the Indian subcontinent. However, in order to uncover the population specific novel polymorphisms and common variations, identification of genetic variations using population level approach is essential. This study presents antigenic repertoires of sexual stage antigens (Pvs25 and Pvs28) of *P. vivax *isolates from different geographical regions of Indian subcontinent.

## Methods

### Study sites and sample collection

Blood samples were collected from five widely separated geographical regions of the Indian subcontinent namely Delhi (2005); Chennai, Tamil Nadu (2005); Kamrup, Assam (2007); Nadiad, Gujarat (2005) and Panna, Madhya Pradesh (2006). Details of epidemiological and geographical information about these study sites are reported elsewhere [21]. Finger prick blood was spotted on autoclaved Whatman filter paper strips (Number 3) from the symptomatic patients in active case detection surveys as well as from patient attending clinics. A total of one hundred microscopically diagnosed *P. vivax *positive (20 from each site) bloods were spotted on autoclaved Whatman filter paper strips (Number 3) and were stored at 4.0°C. This study was approved by the ethics committee of the National Institute of Malaria Research, New Delhi. All bloodspots were collected only after obtaining consent of the patients.

### DNA extraction, PCR, and DNA sequencing

Genomic DNA was extracted from bloodspots using QIAamp mini DNA kit as per manufacturer's instructions. Genomic DNA was eluted in 120.0 μl triple sterile water and store in -20°C until use. One step modified PCR strategy was employed for amplification of *pvs25/pvs28 *reported earlier [20]. PCR products were cleaned up using Exonuclease I/Shrip alkaline phosphates treatment according to manufacturer's instructions. Purified PCR products were outsourced to Macrogen Inc, Korea for DNA sequencing [22]. Each sample was sequenced with both forward and reverse primers. DNA sequences were edited and aligned (ClustalW method) with EditSeq and MegAlign module of DNA Lasergene software version 7.0 (Madison, USA). All sequences have been submitted to the GenBank (HM048519-HM048618, FJ490913-FJ490962 and JF824132-JF824147). All sequences of *pvs25 *and *pvs28 *were compared with Sal-1 reference sequences of accession no. AF083502 and AF083503, respectively for mutation identification.

### Population genetic structure

Population structure of *P. vivax *among five different geographical regions was estimated with pair-wise *F_ST _*difference in population comparison test, using Arlequin software package [23]. This method provides statistical power to view genetic distance among different populations/geographical regions on the basis of frequency distribution of haplotypes.

## Results

*Pvs25 *gene was successfully PCR amplified and sequenced from 100 *P. vivax *isolates collected from five geographical regions (N = 20 from each study site). PCR amplified fragment was 830 bp in length, which covers an upstream non-coding (1-223 bp) and a coding (224-830 bp) region. DNA sequence analysis revealed a total of 16 nucleotide substitutions in both non-coding (4) and coding (12) fragments and an insert of seven nucleotides (TACTTGC) in non-coding fragment. Within the coding region, two nucleotide substitutions were synonymous whereas ten were non-synonymous. All non-synonymous substitutions are listed in Table 1. The domain-wise analysis revealed a single amino acid substitution in EGF-2 (E97K), four in EGF-3 (I130T, Q131K, C137W, and A138G), and five in EGF-4 (E174K, E183K, S196F, S198T and V199E) (Table 1). Among these amino acid substitutions, E97K (EGF-2) and I130T, Q131K (EGF-3) were observed to be polymorphic whereas remaining seven were singletons (mutations observed only in single sequence out of total sequences analysed).

**Table 1:** Pvs25 amino acid substitutions in Indian and global isolates of *Plasmodium vivax*

Geographical regions	SS	EGF-1	EGF-2	EGF-3						EGF-4						References
											
	0 0 2	0 8 7	0 9 7	1 3 0	1 3 1	1 3 2	1 3 7	1 3 8	1 4 9	1 7 0	1 7 4	1 8 3	1 9 6	1 9 8	1 9 9	
Sal-1 strain	N	Q	E	I	Q	S	C	A	K	C	E	E	S	S	V	[14]
Iran	-	Q/K	E/Q	T	-	-	-	-	-	-	-	-	-	-	-	[18]
Turkey	N/D	K	-	T	-	-	-	-	-	-	-	-	-	-	-	#
India	-	-	Q	T	-	-	-	-	-	-	-	-	-	-	-	[16]
Bangladesh	-	-	E/Q	T	K	-	-	-	-	-	-	-	-	-	-	[15]
Thailand	-	-	E/Q	T	Q/K	-	-	-	-	-	-	-	-	-	-	[20]
Indonesia	-	-	Q	T	Q	-	-	-	-	-	-	-	-	-	-	[16]
Viet Nam	N/D	-	-	T	-	-	-	-	-	-	-	-	-	-	-	#
N Korea	-	-	-	T	-	-	-	-	-	-	-	-	-	-	-	[16]
S Korea	N/D	-	E/Q	T	-	-	-	-	-	-	-	-	-	-	-	[19]
PNG	-	-	-	T	K	R	-	-	N	-	-	-	-	-	-	[15]
Mexico	-	Q/K	-	T	-	-	-	-	-	-	-	-	-	-	-	[17]
Honduras	-	-	-	-	-	-	-	-	-	-	-	-	-	-	-	[16]
El Salvador	-	-	-	-	-	-	-	-	-	-	-	-	-	-	-	[16]
Colombia	-	K	-	-	-	-	-	-	-	-	-	-	-	-	-	[16]
Brazil	-	Q/K	-	-	-	-	-	-	-	-	-	-	-	-	-	[16]
Nicaragua	-	-	-	-	-	-	-	-	-	R	-	-	-	-	-	[16]
India	-	-	E/Q	T	Q/K	-	C/W	A/G	-	-	E/K	E/K	S/F	S/T	V/E	*

Bold face values: amino acid substitution position, #: Unpublished data, *: Data of present study

*Pvs28 *was successfully PCR amplified and sequenced from 66 isolates collected from five geographical regions namely Delhi (N = 15), Panna (N = 6), Nadiad (N = 11), Chennai (N = 14), and Kamrup (N = 20). PCR amplified fragment was 650 bp in length and covers coding domains of signal sequence, EGF-1 to EGF-4, and C terminal hydrophobic region (THR). A total of 18 segregating sites and 21 nucleotide substitutions were observed in the study isolates. Among the total nucleotide substitutions, five were synonymous whereas 16 were non-synonymous. The majority of substitutions were singletons in nature (11/21). All non-synonymous substitutions are listed in Table 2. The observed domain wise analysis revealed two amino acid substitutions in a signal sequence (H5T and H5Y), two in EGF-1 (M52L and A53V), four in EGF-2 (T65K, V79E, L98I and E105K), two in EGF-3 (L116V and T140S), two in EGF-4 (G191D and D210G), and four in THR (G212R, S216T, V223L and I224M). Out of 16, eight amino acid substitutions are observed to be novel. The novel amino acid substitutions were mapped in the domains of signal sequence (H5T and H5Y), EGF-2 (V79E), EGF-4 (G191D and D210G), and THR (G212R, S216T and V223L).

**Table 2:** Pvs28 amino acid substitutions in Indian and global isolates of *Plasmodium vivax*

Geographic regions	SS	EFG-1		EGF-2							EGF-3				EGF-4				THR				References
														
	0 0 5	0 5 2	0 5 3	0 6 5	0 7 9	0 8 1	0 9 5	0 9 8	105	1 0 6	110	113	1 1 6	1 4 0	1 5 9	191	Repeats	2 1 0	212	216	2 2 3	224	
Sal-1 strain	H	M	A	T	V	A	G	L	E	V	Y	N	L	T	K	G	6	D	G	S	V	I	[14]
Iran	-	L	-	T/K	-	-	-	-	-	-	-	-	-	S	-	-	4-6	-	-	-	-	-	[18]
India	-	L	-	K	-	-	-	-	-	-	-	-	-	S	-	-	4	-	-	-	-	-	[15]
Bangladesh	-	M/L	-	T/K	-	-	-	L/I	-		N	S	L/V	T/S	K/R	-	5-7	-	-	-	-	I/M	[15]
Thailand	-	M/L	A/V	T/K	-	A/V	G/N	L/I	E/K	V/E	N	-	L/V	T/S	-	-	5-7	-	-	-	-	-	[20]
Mexico	-	L	-	T	-	-	-	-	-	-	-	-	-	S	-	-	5-6	-	-	-	-	-	[17]
S Korea	-	L	-	-	-	-	-	-	-	-	-	-	-	S	-	-	6	-	-	-	-	-	[19]
India	H/T/Y	M/L	A/V	T/K	V/E	-	-	L/I	E/K	-	-	-	L/V	T/S	-	D	3-6	D/G	G/R	S/T	V/L	I/M	*

Bold face values: amino acid substitution position, and *: Data of present study

### Geographical distribution and comparison with global amino acid substitutions

In Pvs25, E97K and I130T were the most common amino acid substitutions observed in isolates of five geographical regions of the Indian subcontinent. The amino acid substitution Q131K was very frequent in isolates of Kamrup region (55.0%), very less in isolate from Panna (5.0%) and Chennai (5.0%), and was absent in isolates of Nadiad and Delhi. Region specific amino acid substitutions were observed more in isolates from Kamrup (C137W, A138G, E174K and S196F) and less in isolates from Delhi (E183K), Panna (S198T) and Nadiad (V199E). None of the amino acid substitution was observed to be specific for isolates of Chennai. Comparison of amino acid substitutions observed in present study with those reported in global isolates suggest that amino acid substitutions in EGF-1 (E97K) and EGF-2 (E97K and Q131K) were present in global as well as in Indian isolates. However, amino acid substitutions found in signal sequence and EGF-1 domains were not observed in Indian isolates. EGF-3 revealed amino acid substitution at codon 132, found only in isolates of Papua New Guinea, was not observed in Indian isolates (Table 1).

In Pvs28, M52L, T65K and T140S were the most common amino acid substitutions observed among five geographical regions of the Indian subcontinent. The seven amino acid substitutions (A53V, V79E, L98I, E105K, L116V, V223L and I224M) were observed to be specific for isolates of Kamrup region, whereas four were specific for Chennai (G191D, D210G, G212R and S216T) and one was for Panna (H5T) and Nadiad (H5Y). None of the region specific amino acid substitutions were observed in isolates of Delhi. The eight amino acid substitutions observed in present study (Table 2) were also reported from the global isolates. Majority of amino acid substitutions observed in isolates of Kamrup region were more identical to the amino acid substitutions found in isolates of Bangladesh and Thailand. On the basis of amino acid substitution, isolates from Nadiad, Panna, Chennai and Delhi did show more similarity compared to the isolates from the north-eastern region (Kamrup) of the country. Several of the amino acid substitutions (Codon 81, 95, 106, 110, 113 and 159) found in global isolates were not observed in the Indian subcontinent.

### Tandem repeat polymorphism

Tandem repeat polymorphism at Pvs28 was observed due to deletion/insertion of five amino acid repeat unit (GEGGS/D) in study isolates from all geographical regions of Indian subcontinent. Four tandem repeat variants observed in present study, were (GEGGS/D)3, (GEGGS/D)4, (GEGGS/D)5, and (GEGGS/D)6. Tandem repeat variants (GEGGS/D)4 and (GEGGS/D)5 were observed in all five geographical regions whereas tandem repeat (GEGGS/D)3 and (GEGGS/D)6 were only observed in isolates of Delhi and Kamrup regions, respectively (Table 3). Tandem repeat variant (GEGGS/D)6 observed in isolates of Kamrup region, was also commonly found in isolates from adjoining countries, such as Bangladesh and Thailand. Among global isolates, 4-7 tandem repeats of GEGGS/D were found, whereas in Indian isolates, an additional variant of (GEGGS/D)3, was observed.

**Table 3:** Tandem repeat polymorphism at Pvs28 and their distribution in different geographical regions of India

Tandem repeats	Distribution of tandem repeat variants (%)				
	
	Delhi	Chennai	Panna	Nadiad	Kamrup
(GEGGS/D)3	13.33	0	0	0	0
(GEGGS/D)4	73.34	50.0	83.33	55.46	5.0
(GEGGS/D)5	13.33	50.0	16.67	54.54	10.0
(GEGGS/D)6	0	0	0	0	85.0
Total (N)	15	14	6	11	20

### Population genetic structure

**Figure 1:**
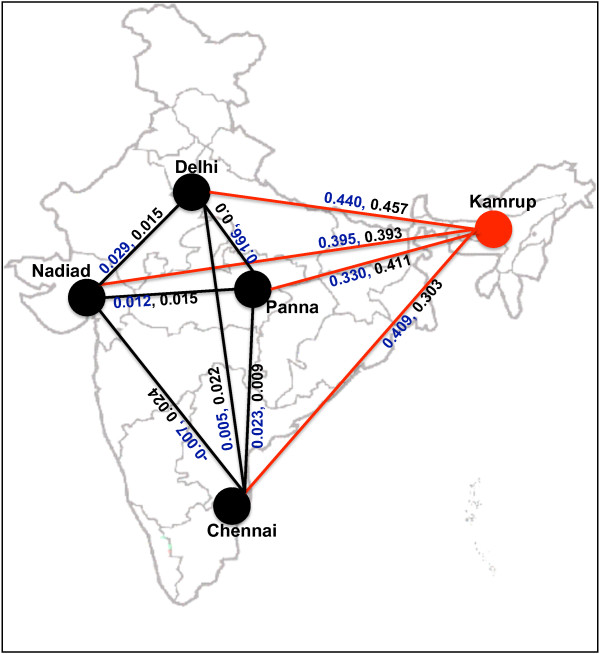
**Genetic differentiation between geographical regions of Indian subcontinent**. Red line colour indicates *Fst *with significant p value (p < 0.001) between populations. Black line indicates *Fst *with non-significant p value (p > 0.05) between populations. Values in black and blue colours represent *Fst *estimated at *pvs25 *and *pvs28 *respectively.

Haplotyping using DNA sequence polymorphism revealed 10 and 15 haplotypes in *pvs25 *and *pvs28*, respectively. Haplotype frequency distribution revealed a single major haplotype at *pvs25 *(Hap1) and *pvs28 *(Hap8) among five geographical regions of the Indian subcontinent (Table 4). Highest number of haplotypes was observed in isolates of Kamrup (north-eastern region). All though, few haplotypes observed in relatively low frequency at *pvs25 *(9) and *pvs28 *(14) were specific to a particular population. Pair-wise comparison using *Fst *difference between populations is presented in Figure 1. The pair-wise *Fst *difference on the basis of both *pvs25 *and *pvs28 *haplotypes, revealed that all studied populations significantly differ with Kamrup population (north-eastern region) with a non-significant differentiation among the four regions (Panna, Delhi, Nadiad, and Chennai) (Figure 1). The *F_ST _*values showed existence of significant level of gene flow among isolates of Chennai, Delhi, Panna, and Nadiad regions.

**Table 4:** Frequency distribution of haplotypes at *pvs25 *and *pvs28 *among Indian subcontinent

*Pvs25*	Geographic location				
	
Haplotype	Delhi (N = 20)	Nadiad (N = 20)	Panna (N = 20)	Chennai (N = 20)	Kamrup (N = 20)
**Hap1**	**0.95**	**0.85**	**0.90**	**0.95**	**0.30**
Hap2	0	0	0	0.05	0.35
Hap3	0.05	0	0	0	0
Hap4	0	0.10	0	0	0
Hap5	0	0.05	0	0	0
Hap6	0	0	0.05	0	0.15
Hap7	0	0	0	0	0.05
Hap8	0	0	0	0	0.05
Hap9	0	0	0	0	0.10
Hap10	0	0	0.05	0	0
***Pvs28***	(N = 15)	(N = 11)	(N = 6)	(N = 14)	(N = 20)
Hap1	0	0	0	0	0.05
Hap2	0	0	0	0	0.05
Hap3	0	0	0	0	0.05
Hap4	0	0	0	0	0.05
Hap5	0	0	0	0	0.05
Hap6	0	0	0	0	0.10
Hap7	0	0	0	0	0.10
**Hap8**	**1**	**0.91**	**0.83**	**0.85**	**0.25**
Hap9	0	0	0	0	0.10
Hap10	0	0	0	0	0.05
Hap11	0	0	0	0	0.15
Hap12	0	0	0.16	0	0
Hap13	0	0	0	0.07	0
Hap14	0	0	0	0.07	0
Hap15	0	0.09	0	0	0

## Discussion

The worldwide spread of drug resistant *Plasmodium *species compounding the lack of anti-malarial vaccine in near future calls for identifying the new vaccine/drug targets in order to design more effective anti-malarial control measures. The excellent capability of parasite for generating polymorphism for its survival is the major challenging task for designing anti-malarial vaccine. Pvs25 and Pvs28 are the two promising candidates for the development of anti-malarial transmission blocking vaccine [12]. The antigenic diversity reported in target molecules (Pvs25 and Pvs28) could hinder the efficacy of vaccine [14,18,20]. Therefore, identifying sequence variations in transmission-blocking vaccine candidates may be helpful in designing an effective anti-malarial vaccine.

A previous study has identified limited polymorphism in both Pvs25 and Pvs28 in *P. vivax *clinical isolate collected from a Japanese tourist, who had acquired infection from India. In contrast, present prospective study identified many additional amino acid substitutions in Indian subcontinent at both Pvs25 and Pvs28 (Table 1 and 2). Indian isolates revealed 10 and 15 amino acid substitutions at Pvs25 and Pvs28, respectively, which is more than to that observed among global isolates. This suggests that mutations in both sexual stage antigen genes are not limited, as previously it was reported. More than 50% of the major amino acid substitutions observed at Pvs25 and Pvs28 in global isolates were shared by Indian isolates which suggests that Indian isolates harbours major pool of global sexual stage antigenic repertoires.

Comparison of amino acid substitutions at both Pvs25 and Pvs28 suggests that majority of the substitutions observed in Indian isolates were similar to the amino acid substitutions reported in isolates from Thailand and Bangladesh. All though, sequence polymorphism at Pvs25 & Pvs28 was investigated using limited number of isolates from Bangladesh [15], and Southeast Asia [15,16,20]. The present data suggests that by increasing sample size from these countries, it could be possible to uncover much additional amino acid substitution. The higher number of amino acid substitutions observed in present study compared with global isolates could be due to large samples analyzed, which were collected from five widely separated geographical regions of India.

Regardless of many haplotypes observed in present study, the major haplotype appeared to be single at both Pvs25 & Pvs28 (Table 4) in four geographic regions (Delhi, Panna, Nadiad and Chennai). Similarly, the *Fst *analysis suggests that genetic structure of parasite present in north-eastern region is different than remaining part of the Indian subcontinent. The amino acid substitutions and tandem repeat polymorphism data collectively suggests that isolates from north-eastern region have more similarity with the isolates from Bangladesh and Thailand. This is strongly supported with a recent study that suggests a higher frequency of quadruple mutant *pvdhfr *genotype (61.9%) in north-eastern region [24]. The frequency of quadruple mutant *pvdhfr *genotype is rarely observed in remaining part of the country, however it is a major genotype in isolates of Myanmar (71.0%) and Thailand (96.0%) [25,26]. The higher genetic identity of *P. vivax *isolates from north-eastern region with the isolates from Bangladesh and Thailand could be due to 1) similarity in malaria transmitting vector species (*Anopheles minimus *and *Anopheles dirus*) [27] due to similar geographical environment, and 2) as north-eastern states are surrounded by international borders and frequent migration of peoples is possible due to porous borders. These above factors may contribute the inflow of malaria parasites in adjoining north-eastern region of the country.

This study added seven and eight novel amino acid substitutions in Pvs25 and Pvs28 respectively suggesting the importance of population level approach to identify amino acid substitutions in vaccine candidates. The total number of global substitutions increases from 8 to 15 at Pvs25 and 14 to 21 at Pvs28. The increase in number of amino acid substitutions at the two sexual stage antigens may hampers effectiveness of Sal 1 strain based transmission-blocking vaccine [12] in different geographical regions of the globe. Though, one study has evaluated similar efficacy of Sal-1 strain based vaccine for variants sequence observed in Thai isolates that suggest that Sal-1 strain based vaccine could have wider usability [20]. Since, most of the amino acid substitutions observed in Indian isolates were similar to those observed in isolates of Thailand, therefore, one could expect that Sal-1 strain based transmission-blocking vaccine could work in Indian geographic regions. However, for a better success of transmission-blocking vaccine, evaluation of the Sal-1 strain based vaccine for all major haplotypes observed in Indian and global isolates may be warranted.

In conclusion, present study uncovered several new amino acid substitutions at both Pvs25 and Pvs28 among Indian isolates and the most frequent amino acid substitutions observed, were shared by global isolates. *P. vivax *isolates from north-eastern region are different from remaining part of Indian subcontinent and have high similarity with the isolates from Bangladesh and Thailand. The amino acid substitution data of present study would be helpful in designing a more appropriate effective anti-malarial transmission-blocking vaccine.

## Competing interests

The authors declare that they have no competing interests.

## Authors' contributions

SKP carried out the experiment design, experimental work, data analysis, and manuscript writing, HJ conceived and coordinated the study, and VKD supervised overall work and contributed in data analysis and manuscript writing. All authors read and approved the final manuscript.
